# Transatlantic Telerobotic Coronary Angiography: A Pre-clinical Feasibility Study

**DOI:** 10.1016/j.jacadv.2024.101456

**Published:** 2024-12-16

**Authors:** Ryan D. Madder, Stacie VanOosterhout, Jessica L. Parker, Alessandro Candreva

**Affiliations:** a Frederik Meijer Heart & Vascular Institute, Corewell Health West, Grand Rapids, Michigan, USA; b Department of Cardiology, University Heart Centre, University Hospital Zürich, Zürich, Switzerland

**Keywords:** cardiac catheterization, robotic, telemedicine, telesurgery

## Abstract

**Background:**

Patients in many underserved geographies lack access to invasive coronary angiography (ICA).

**Objectives:**

This preclinical study explored the feasibility of telerobotic ICA between separate continents.

**Methods:**

Using a novel robotic system, attempts were made to navigate a magnetic guidewire and diagnostic catheter from the aortic arch into a target coronary artery ostium in a fluid-filled cardiac model. The model and robotic system were located in Zürich, Switzerland. The operating physician was either “onsite” in the laboratory in Zürich or “transatlantic,” in which the physician remotely controlled the robot from Grand Rapids, Michigan, USA. An onsite control group of 40 manual catheterization cases was made for comparison. The primary endpoint was technical success (catheter engagement into target ostium without conversion to manual). A secondary endpoint was engagement time (time from initial robotic manipulation to engagement in target ostium).

**Results:**

In 260 consecutive attempts, of which 40 (15.4%) were “onsite” and 220 (84.6%) were “transatlantic,” technical success was 97.5% onsite and 100% in the transatlantic group (*P* = 0.154). Median engagement times were 33.2 seconds (25th, 75th percentile: 24.9, 45.0 seconds) onsite and 26.7 seconds (25th, 75th percentile: 21.7, 35.5 seconds) transatlantic (*P* = 0.003). Median engagement time was faster for manual cases (17.1 seconds [25th, 75th percentile: 12.2, 23.2 seconds]) compared to both robotic groups (*P* < 0.001).

**Conclusions:**

In this preclinical study, the proof-of-concept for telerobotic ICA was successfully demonstrated. Furthermore, the current limits of telerobotic capabilities were tested by conducting ICA between separate continents and showing that transatlantic telerobotic navigation of endovascular devices is now technically possible.

Robotic systems for performing percutaneous coronary intervention (PCI) have been in clinical use for more than a decade.^1^^,^^2^ Capable of performing PCI with high technical and procedural success in both simple and complex coronary lesions,^1^, ^2^, ^3^ endovascular robotic systems have more recently been adapted and studied for use in telerobotic applications.^4^, ^5^, ^6^, ^7^, ^8^ Prior research has demonstrated the technical success of telerobotic PCI in in vitro models,^5^^,^^6^ in vivo animal models,^5^^,^^7^ and in humans.^8^ The interest in further developing telerobotic PCI capabilities lies in ongoing geographical disparities in PCI access and from evidence that the lack of PCI availability among patients with myocardial infarction associates with higher mortality.^9^^,^^10^

When considering the further development of telerobotic PCI, it is crucial to note that all patients undergoing PCI first undergo invasive coronary angiography (ICA) to define coronary anatomy and identify the presence and location of culprit lesions. Consequently, developing telerobotic ICA may be integral to the further development of telerobotic PCI, but in all previous studies of telerobotic PCI, the guide catheter was manually inserted into the target coronary ostium by bedside clinical personal.^5^, ^6^, ^7^, ^8^ Robotic ICA, in which a robotic system is used to navigate catheters from a position in the aorta to the target coronary ostium, can be performed successfully in clinical settings^11^ but has only been evaluated with the physician in the same room as the patient. To our knowledge, telerobotic ICA has not been previously studied. This preclinical study was conducted to: 1) demonstrate the proof-of-concept for telerobotic ICA using a novel robotic system designed for the electromagnetic navigation of endovascular devices; 2) test the current limits of telerobotic capabilities by conducting the procedures between separate continents; and 3) explore whether it is possible for an operating physician to perform telerobotic endovascular procedures from multiple settings outside of a hospital campus.

## Methods

### Robotic system

**Figure 1:**
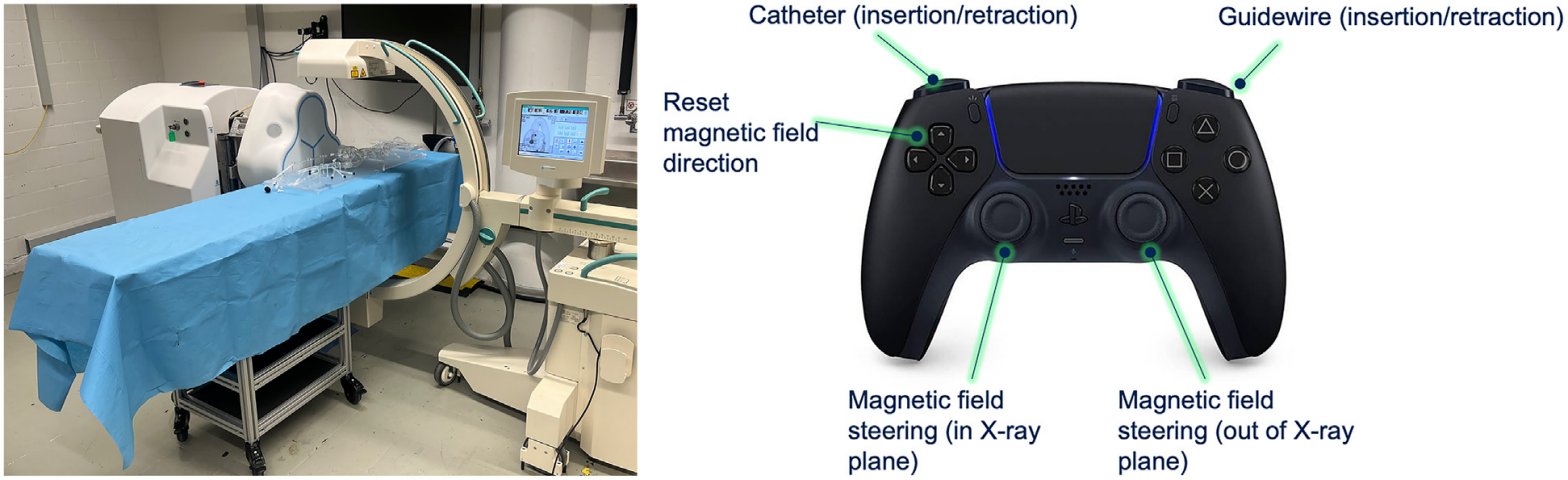
**Novel Electromagnetic Robotic System** (Left) Shown is the novel robotic System (Located on far Side of Procedure Table) Designed for Electromagnetic Navigation of Endovascular Devices. (Right) Shown is the controller used in this study and a brief description of the functionality of the various buttons.

This study was performed using a novel robotic system (Electromagnetic Navigation System, Nanoflex Robotics AG) designed for the electromagnetic navigation of endovascular devices.^12^ The system is comprised of a bedside magnetic field generator, bedside robotic drive, and robotic controller (Figure 1). The mobile magnetic field generator, consisting of 3 electromagnets arranged in triangular formation, projects a magnetic field onto the patient. The position of the magnetic field generator can be moved to project the field on the desired target anatomy (intracranial, cardiac, peripheral, etc) and its height can be adjusted according to operating table height. The vector of the magnetic field, which is manipulated by handheld robotic controller, can be changed to alter the tip shape and orientation of a 0.035-inch magnetic guidewire having a soft flexible tip. Manipulation of the magnetic field by the robotic controller thereby enables highly dexterous, real-time steering of the magnetic guidewire tip. A graphical user interface displays the magnetic field vector, as well as live fluoroscopic images and a video feed from the procedure room. The motorized robotic drive is capable of advancing and retracting the guidewire and associated devices, including catheters and microcatheters. For the purposes of this study, the robotic system was controlled using a handheld gaming controller (PS5, Sony Interactive Entertainment) having buttons programmed to operate the direction of the magnetic field vector and robotic drive. Since the study was performed in vitro and did not involve human subjects, approval by the local institutional review board was not required.

### In vitro coronary angiography model

This study utilized a fluid-filled silicone model (Neuro Vascular System I, Trando 3D Medical Technology) of the heart and arterial vasculature. Fluoroscopic imaging of the model was performed using a mobile C-arm (Ziehm Vision FD, Ziehm Imaging). Femoral and radial arterial access sites in the model were utilized for testing. For each robotic case, a 0.035-inch magnetic guidewire and a soft, nonangled 5-F diagnostic catheter were manually advanced to the aortic arch and then loaded onto the robotic drive. All subsequent manipulations of the guidewire and catheter were performed robotically under fluoroscopic guidance. At the start of each attempt to engage the coronary arteries, the guidewire was robotically navigated to the ostium of the target coronary artery and then advanced a short distance into the target artery. Thereafter, the diagnostic catheter was robotically advanced over the guidewire into the target coronary artery ostium. Cases were also performed in a manual control group, in which a standard 0.035-inch J-tipped guidewire and pre-shaped 5-F diagnostic catheters were used to engage the target coronary artery ostium using standard clinical techniques.

### Location of the operating physician

For all cases, the cardiac model, magnetic field generator, and robotic drive were colocated in a laboratory in Zürich, Switzerland. For cases performed in the manual control group, an interventional cardiologist (A.C.) stood adjacent to the cardiac model. Robotic cases were characterized as either “onsite,” in which an interventional cardiologist (A.C.) controlled the robotic procedures from the control room of the laboratory in Zürich, Switzerland or “transatlantic,” in which an interventional cardiologist (R.D.M.) controlled the robot in Zürich while located in Grand Rapids, Michigan, USA. Neither the onsite nor the transatlantic interventional cardiologist had any experience operating the robotic system prior to the day of testing. After being provided with verbal instructions on its functionality, both cardiologists were given 20 minutes to practice operating the robot prior to the start of testing. In order to test the ability to perform telerobotic procedures from multiple different locations, transatlantic cases were performed with the interventional cardiologist in one of the 5 locations: 1) campus of a quaternary referral hospital; 2) outpatient medical office building; 3) a coffee shop; 4) physician’s home; and 5) passenger seat of a parked car in a public park.

### Network connectivity

For onsite procedures conducted from the control room in Zurich, the robotic controls communicated with the robotic system over a local network. For transatlantic procedures, the handheld robotic controls were physically connected to a single laptop computer at the control site in the United States which communicated to the robotic system in Switzerland using a websocket over public internet. The websocket enables bi-directional communication over a single, long-lived communication session between the control laptop and robotic system. For transatlantic procedures controlled from the hospital campus, connectivity of the control laptop to the network was studied using Ethernet and the hospital’s local WiFi. Transatlantic procedures performed from the medical office building utilized WiFi and 5G wireless connectivity. Transatlantic procedures conduced from the physician’s home were performed over WiFi. 5G wireless connectivity was utilized for transatlantic procedures performed from the coffee shop and the parked car. The same service provider (Verizon Wireless) was used for all cases performed over 5G wireless.

### Primary and secondary endpoints

The primary endpoint for this study was technical success, defined as the successful movement of devices by the robotic system to engage a catheter into the target coronary ostium without conversion to a manual procedure. Secondary endpoints included catheter engagement time, network latency, perceived latency score, and latency impact score. Engagement time was defined as the time from initial manipulation of the guidewire and catheter to engagement of the catheter into the target coronary ostium. Latency, defined as the network latency for robotic data packet transfer, was recorded at a frequency of 15 Hz and was reported as the mean latency over each set of attempts to engage the coronary ostium. The perceived latency score was graded by the interventional cardiologist after each attempt and was scored on a 5-point Likert scale (5 = imperceptible; 4 = noticeable but minor; 3 = noticeable; 2 = noticeable and major; 1 = unacceptable).^13^ Similarly, latency impact score was graded by the interventional cardiologist after each attempt (5 = no impact; 4 = minor impact but acceptable performance; 3 = noticeable impact, loss in efficiency, but successful outcome; 2 = significant degradation, can complete procedure, but not desirable; 1 = unacceptable).^13^

### Statistical analysis

Histograms of continuous data were visually inspected for normality. Normally distributed continuous variables are shown as mean ± SD. Non-normally distributed continuous variables are shown as median [(25th, 75th percentile). Categorical variables are shown as count (% frequency).

Technical success rates were compared among onsite and transatlantic cases using Fishers Exact test. Onsite robotic engagement times were compared to those of the manual control group using Wilcoxon Rank Sum test. Similarly, Wilcoxon Rank Sum test was used to compare transatlantic robotic engagement times to onsite robotic engagement times. Kruskal Wallis methodology was used to analyze median engagement times across groups according to the location of the operating physician. Kruskal Wallis methodology was similarly used to analyze median engagement times across telerobotic groups according to whether Ethernet, WiFi, or 5G wireless was utilized for network connectivity. When using Kruskal Wallis, if the the overall *P* value was significant, then Wilcoxon Rank Sum was used with a Bonferroni correction to determine where the differences exist. Median engagement times were compared among cases performed using radial versus femoral access using Wilcoxon Rank Sum Test. Median latency values for telerobotic cases performed using Ethernet, WiFi, and 5G wireless connectivity were compared using Wilcoxon Rank Sum Test. Comparison of Perceived Latency Scores and Latency Impact Scores across these groups were compared using Fishers Exact Test. All statistical analyses were generated using SAS (SAS Enterprise Guide software, version 7.1; SAS Institute, Inc).

## Results

Using electromagnetic robotic navigation, a total of 260 attempts were made to engage a diagnostic catheter into a target coronary artery. Of these, 40 (15.4%) attempts were made onsite, in which a physician operator controlled the robotic system onsite from the laboratory in Zürich, and 220 (84.6%) were transatlantic, in which a physician in the United States remotely controlled the robotic system in Switzerland. Among the 40 onsite robotic cases, radial access was used in 20 (50.0%) and femoral access was used in 20 (50.0%). Among transatlantic cases, radial and femoral access were used 140 (63.6%) and 80 (36.4%) cases, respectively. Of the 220 transatlantic attempts, 80 (36.4%) were performed from a hospital campus, 80 (36.4%) from a medical office building, 20 (9.1%) from the physician’s home, 20 (9.1%) from a coffee shop, and 20 (9.1%) from the passenger seat of a parked car (Central Illustration). A control group consisting of 40 manual attempts (20 radial access; 20 femoral access) to engage diagnostic catheters into the target arteries using standard clinical techniques were also made for comparison.

**Central Illustration:**
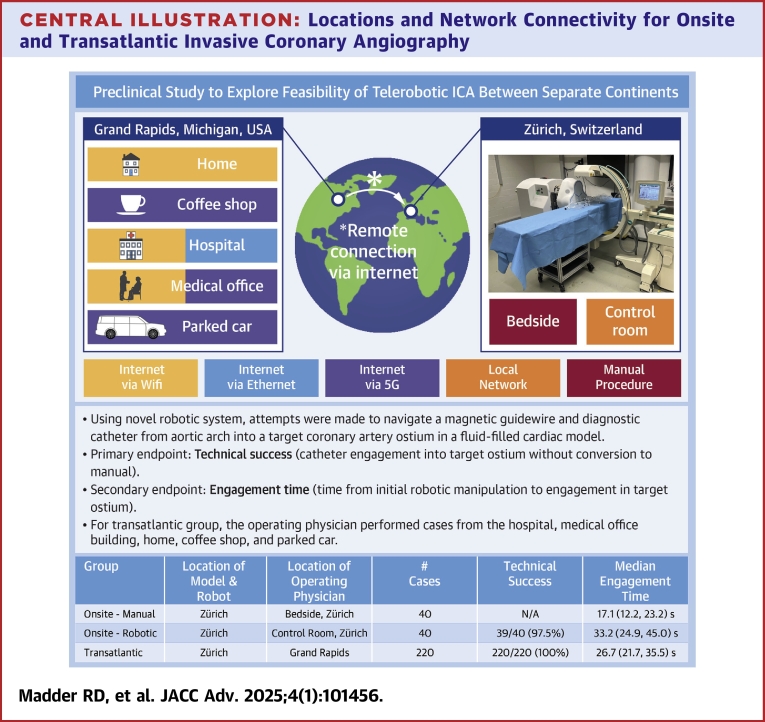
**Locations and Network Connectivity for Onsite and Transatlantic Invasive Coronary Angiography** For all cases, the cardiac model and robotic system were co-located in a laboratory in Zürich, Switzerland. Cases in the Manual Control Group Were Performed by a Bedside IC. Onsite robotic cases were controlled by an IC from the control room in the laboratory in Zürich using the local network. For all transatlantic cases, an IC controlled the robot in Zürich from Grand Rapids, Michigan, USA. The locations From which transatlantic cases were controlled, as well as the type of network connectivity utilized, are represented on the left side of the image. IC = Interventional Cardiologist.

### Technical success

The diagnostic catheter was successfully navigated by the robotic system into the target coronary ostium without conversion to a manual procedure in 259 of 260 (99.6%) robotic attempts. Technical success, achieved in 39 of 40 (97.5%) onsite attempts and in all 220 (100%) transatlantic attempts, was not significantly different between groups (*P* = 0.154). The single onsite case in which technical success was not achieved was the first attempted case. A demonstration of transatlantic electromagnetic robotic navigation of the guidewire and catheter into the right and left coronary arteries is shown in Video 1.

### Engagement time

The median engagement time for electromagnetic robotic navigation of a catheter into the target coronary artery was 33.2 seconds (25th, 75th percentile: 24.9, 45.0 seconds) when performed onsite and was 26.7 [21.7, 35.5] s in transatlantic cases (*P* = 0.003). Median engagement time was faster for manual cases (17.1 seconds [25th, 75th percentile: 12.2, 23.2 seconds] than for both robotic groups (*P* < 0.001). Median robotic engagement time according to vascular access site was 24.8 seconds (25th, 75th percentile: 20.1, 32.7 seconds) for radial access and was 29.8 seconds (25th, 75th percentile: 22.2, 40.4 seconds) for femoral access (*P* = 0.018).

Transatlantic telerobotic catheter engagement times are summarized according to the location of physician operator in Table 1. Significant differences were identified according to the location of robotic controller (*P* < 0.001), although the magnitude of differences was small between groups. Transatlantic telerobotic catheter engagement times according to the type of network connection utilized are shown in Table 2. Although significant differences were identified across groups (*P* < 0.001), the magnitude of the differences was small.

**Table 1:** Transatlantic Technical Success and Catheter Engagement Times According to Operating Physician Location

	Hospital (n = 80)	Office (n = 80)	Home (n = 20)	Coffeeshop (n = 20)	Car (n = 20)	*P* Value
Technical success	80 (100)	80 (100)	20 (100)	20 (100)	20 (100)	N/A
Engagement time (s)	32.8 (25.6, 45.7)	26.8 (21.7, 32.6)	22.6 (20.7 26.5)	19.8 (18.9, 24.0)	22.6 (20.5, 27.1)	<0.001

Values are n (%) or median (25th, 75th percentile).

**Table 2:** Transatlantic Telerobotic Metrics According to Type of Network Connectivity

	Ethernet (n = 40)	WiFi (n = 100)	5G Wireless (n = 80)	*P* Value
Latency (ms)	134 (134, 134)	143 (118, 151)	142 (141, 145)	0.20
Perceived Latency Score				
1	0 (0)	0 (0)	0 (0)	
2	0 (0)	0 (0)	0 (0)	
3	31 (77.5)	24 (24.0)	10 (12.5)	<0.001
4	9 (22.5)	72 (72.0)	70 (87.5)	
5	0 (0)	4 (4.0)	0 (0)	
Latency Impact Score				
1	0 (0)	0 (0)	0 (0)	
2	0 (0)	0 (0)	0 (0)	
3	0 (0)	0 (0)	0 (0)	0.006
4	3 (7.5)	0 (0)	0 (0)	
5	37 (92.5)	100 (100)	80 (100)	

Values are n (%) or median (25th, 75th percentile).

### Transatlantic latency

No significant differences in median latencies were identified between cases performed over Ethernet, WiFi, or 5G wireless connectivity (Table 2), with a median latency of <150 ms in all 3 groups. Perceived Latency Scores and Latency Impact Scores are summarized in Table 2. The latency was deemed perceptible (perceived latency score <5) by the physician operator in 100% of cases performed over Ethernet and 5G wireless and in 96% of cases performed over WiFi. When using 5G wireless or WiFi connectivity, the latency was graded as “noticeable but minor” (Perceived Latency Score = 4) in 87.5% and 72.0% of cases, respectively. In 77.5% of cases using Ethernet connectivity, the perceived latency was graded as “noticeable” (Perceived Latency Score = 3). Despite the high rate of perceived latency across all 3 types of network connections, the latency impact score was 5 (“no impact”) in 100% of 5G wireless and WiFi cases and in 92.5% in Ethernet cases. Among the Ethernet cases in which the perceived latency impacted the procedure, the impact was graded as “minor impact but acceptable performance” (Latency Impact Score = 4).

## Discussion

This study makes 3 principal observations relevant to the further development of telerobotic endovascular procedures. First, the technical success of using electromagnetic navigation to telerobotically maneuver a diagnostic catheter from the aortic arch to a target coronary artery was demonstrated in 100% of attempts, was successful from both radial and femoral access sites, and was not found to be significantly different compared to robotic cases performed onsite. Although injection of contrast was not performed, the successful positioning of the diagnostic catheter in the target coronary artery in 220 consecutive telerobotic cases provides evidence for the proof-of-concept that telerobotic ICA is technically possible. Second, the present study tested the limits of contemporary telerobotic capabilities by successfully conducting the procedures in a transatlantic manner in which the coronary model and robot were located in Switzerland and the operating physician and robotic controls were located >4,000 miles away in the United States. Extending the findings of our previous preclinical work in which telerobotic control was demonstrated across the North American continent (6), the present study demonstrates that endovascular devices can be consistently and reliably navigated between separate continents. Third, this study evaluated whether a physician could remotely control a telerobotic device from multiple locations outside the hospital setting and demonstrated a technical success rate of 100% with the physician performing telerobotic navigation of devices from the office, home, a car, and a public space. Whereas conducting procedures from these locations is unconventional, the clinical relevance of this novel observation relates to the combined facts that it is critically important to rapidly treat many cardiovascular emergencies and that the physician responsible for treating the emergency may not always be in close proximity to the hospital. Finally, it is important to note that the novel observations demonstrated in this study, although representing an incremental step forward in the development of telerobotic endovascular capabilities, were limited to performance in a preclinical model. Further study will be needed to see if these promising results can be replicated in vivo.

### Healthcare disparities and the promise of telerobotics

The impetus to develop telerobotic endovascular procedures originates in ongoing geographic disparities in the treatment of patients with myocardial infarction^9^^,^^10^ and stroke.^14^ In the contemporary era, patients hospitalized with acute myocardial infarction at rural hospitals in the United States undergo ICA and PCI at rates significantly lower than those at urban hospitals.^9^ In many underdeveloped regions of the world, barriers to accessing ICA and PCI remain widespread and associate with higher mortality.^10^^,^^15^ Disparities in access to state-of-the-art stroke care are perhaps greater than those for myocardial infarction and are highlighted by data showing almost half the US population cannot reach a thrombectomy-capable center within 1 hour of stroke onset.^3^ This lack of access to thrombectomy is problematic since endovascular thrombectomy is superior to thrombolytic therapy for acute large vessel stroke and has significant implications on long-term disability rates.^16^

Telerobotic procedures have emerged as a potentially viable strategy to address geographic disparities in healthcare. The findings of the present study, which is the first to investigate telerobotic ICA, are in line with prior studies of telerobotic PCI in in vitro models,^5^^,^^6^ animals,^5^^,^^7^ and in humans,^8^ and with prior studies of telerobotic manipulation of neurovascular devices in in vitro models^17^ and cadavers.^18^ In prior telerobotic PCI studies, the guide catheter was manually inserted into the target coronary ostium by bedside personnel prior to attempting telerobotic PCI. The present telerobotic results, as well as those of a prior onsite clinical study of robotic ICA,^11^ demonstrate that catheters can be successfully navigated to engage a target artery using a robotic system. Furthermore, the present study expands the findings of these prior studies by extending the reach of telerobotic endovascular procedures from one continent to another. The observation in this study that endovascular devices can be successfully telerobotically navigated across the Atlantic is consistent with the observations of several recent studies of telesurgeries performed over vast distances using 5G networks^19^^,^^20^ and with the first transatlantic telesurgery in 2001 in which a surgeon in New York successfully performed a telerobotic laparoscopic cholecystectomy on a patient in France.^21^

Despite the current and prior studies having successfully demonstrated the technical capabilities to perform telerobotic endovascular procedures in vitro and in vivo, it is important to note that adoption of telerobotic procedures into routine clinical practice has not yet occurred. The reasons accounting for this lack of uptake include multiple human and technological factors. Perhaps the greatest human factor previously limiting telerobotic adoption was a general lack of acceptance of telemedicine among both patients and physicians. Out of necessity, the COVID-19 pandemic rapidly pushed telemedicine into routine practice thereby accelerating an acceptance of telemedicine among patients and physicians. It is possible that this greater acceptance of telemedicine in general may eventually extend to telerobotic procedures. Among several technological limitations previously preventing the adoption of telerobotics was a lack of widespread availability of ultrafast network connectivity over which telerobotic surgeries could be safely conducted. The recent emergence of 5G wireless technology will likely overcome this latter barrier to telerobotic adoption.

### Contemporary networks and latency

The ability to perform endovascular telerobotics between separate continents is enhanced by the fast performance and extensive bandwidth of contemporary networks, including 5G wireless technology. In the present study, the robotic commands generated by the robotic controls in the United States reached the robot in Switzerland with a latency of <150 ms regardless of whether Ethernet, WiFi, or 5G wireless connectivity was utilized. This latency is well below the 400 ms threshold previously demonstrated to be associated with the perception of latency by physicians during robotic PCI procedures.^7^^,^^13^ However, latency was perceived by the operating physician in nearly all telerobotic cases in this study, likely attributable to the unmeasured latency of transmitting live fluoroscopic images from the laboratory in Switzerland to the control site in the United States. Although frequently perceived, the latency was deemed by the physician operator as having no or minor impact in nearly all procedures. Future studies will determine if enhancement in network performance will ultimately facilitate telerobotic procedures over vast distances with no perceived latency.

### Electromagnetic device navigation

This study was conducted with a novel endovascular robotic system utilizing electromagnetic navigation of devices. Magnetic robotic catheter navigation is not new, as it has been used in clinical electrophysiology procedures for 2 decades^22^ but differs from other endovascular robotic systems which steer devices through advancement, retraction, and torquing.^1^^,^^2^ One major advantage of using electromagnetic device navigation is the ability to change the tip shape of an indwelling wire in real time. For onsite procedures performed in this study, the time required to robotically navigate a catheter into the target coronary artery was statistically significantly slower than the manual approach, but the observed differences in engagement times between robotic and manual procedures are unlikely to be clinically significant. Furthermore, transatlantic telerobotic engagement times compared favorably to those performed onsite, with a median transatlantic engagement time of <30 seconds per case. Although significant differences in onsite and transatlantic engagement times were observed, the clinical significance of the small magnitude of the differences is likely negligible.

### Study Limitations

The in vitro design of the present study represents a limitation and future studies performed in vivo will be required to confirm the findings. The study was also limited considering contrast injection, which is required for ICA to be completed, was not actually performed after catheter engagement. When performing telerobotic ICA in the future, contrast injection will likely be performed by the onsite team, as was done in previous studies of telerobotic PCI.^4^^,^^5^^,^^7^^,^^8^ The study is further limited considering telerobotic PCI was not attempted following telerobotic catheter engagement. The latency of fluoroscopic image transfer was not measured, thereby representing another limitation. The learning curve of electromagnetic robotic device navigation, which likely impacted the observed results, was not studied. The execution of the onsite and transatlantic procedures by 2 different operators represents a significant limitation and likely introduced some inter-operator variability, which will need to be further evaluated in a future study. Finally, the concept of using telerobotic technology to eliminate healthcare disparities still has multiple barriers limiting widespread adoption, including the significant costs associated with robotic systems, the need for trained bedside personnel, and regulatory and legal hurdles. Additional work is required to address these barriers.

## Conclusions

Using a novel robotic system designed for the electromagnetic navigation of endovascular devices, this preclinical study demonstrated the technical success of telerobotically navigating a diagnostic catheter to a target coronary artery and thereby provides evidence for the proof-of-concept of telerobotic ICA. This study also tested the limits of telerobotic capabilities by conducting the procedures between separate continents and showed that consistent and reliable transatlantic telerobotic navigation of endovascular devices is now technically possible. These observations may have future implications on addressing geographic disparities in access to endovascular procedures.

Perspectives **Competency in Medical Knowledge**

Robotic systems for performing PCI have been in clinical use for more than a decade, are capable of performing PCI with high technical and procedural success in both simple and complex coronary lesions, and have been previously studied in the performance of telerobotic PCI. This preclinical study demonstrated the technical success of telerobotic invasive coronary angiography, tested the limits of contemporary telerobotic capabilities by conducting the procedures between separate continents, and showed that consistent and reliable transatlantic telerobotic navigation of endovascular devices is now technically possible.

**Translational Outlook**

Future studies are needed to replicate the present results *in vivo* and to determine if telerobotics can be applied to address existing geographic disparities in access to endovascular procedures.

## Funding support and author disclosure

This study was supported by Nanoflex Robotics AG, Zürich, Switzerland. Dr Madder has received speaker honoraria from Abbott Vascular, Boston Scientific, Corindus, and Infraredx; has served as a consultant to Abbott Vascular, Angiowave Imaging, Corindus, Infraredx, Nanoflex Robotics, RapidAI, and Spectrawave; has received research support from Angiowave Imaging, Corindus, 10.13039/100018503Infraredx, Microbot Medical, and Nanoflex Robotics; and serves on the advisory boards of Gentuity, Medtronic, and Spectrawave. Dr Candreva has consultancy agreements with Medyria AG and Nanoflex Robotics AG. All other authors have reported that they have no relationships relevant to the contents of this paper to disclose.

## Supplementary data

Video 1 Example of Transatlantic Telerobotic Electromagnetic Navigation. This video shows transatlantic telerobotic electromagnetic navigation of a magnetic guidewire and catheter into the left coronary artery. The cardiac model and robot were located in Zürich, Switzerland. In this video, the operating physician performed the robotic procedure from the passenger seat of a parked car in Grand Rapids, Michigan, USA. Shown are corresponding fluoroscopic images (left) and real-time manipulation of the magnetic field vector (right).
